# Adsorption of Pb(II) from Aqueous Solution by Mussel Shell-Based Adsorbent: Preparation, Characterization, and Adsorption Performance

**DOI:** 10.3390/ma14040741

**Published:** 2021-02-05

**Authors:** Quan Wang, Fangyuan Jiang, Xiao-Kun Ouyang, Li-Ye Yang, Yangguang Wang

**Affiliations:** School of Food and Pharmacy, Zhejiang Ocean University, Zhoushan 316022, China; wqzjou@163.com (Q.W.); jfy0925@163.com (F.J.)

**Keywords:** calcined mussel shell powder, adsorption capacity, Pb(II), isotherm

## Abstract

As a natural biological adsorbent, shell powder is inexpensive, highly efficient, and does not leave any chemical residue; thus, it can be used to remove contaminants from water. In this study, we used mussel shells as a raw material to prepare an adsorbent. Scanning electron microscopy was used to observe the surface morphology of the mussel shell powder before and after calcination, and X-ray diffraction measurements, Fourier transform infrared spectroscopy, differential scanning calorimetry, X-ray photoelectron spectroscopy, and Brunauer–Emmett–Teller measurements were performed to analyze the structure and composition of calcined mussel shell powder. Characterization of the shell powder before and after calcination revealed a change from calcium carbonate to calcium oxide, as well as the formation of a surface porous structure. Using Pb(II) as a representative contaminant, various factors affecting the adsorption were explored, and the adsorption mechanism was analyzed. It was found that the adsorption is consistent with the Freundlich adsorption isotherm and the pseudo second-order model. The calcined mussel shell powder exhibits excellent adsorption for Pb(II), with an adsorption capacity reaching 102.04 mg/g.

## 1. Introduction

With the rapid growth of the global population and economy, in addition to gradual improvements in the quality of life, the demand for healthy food has increased continuously in recent years [1]. Although consumers prefer to purchase aesthetically-pleasing fruits and vegetables, the majority of these products have been grown using pesticides or preservatives that can remain in the soil, damage soil quality [2], and leave residues on the crops thereby resulting in bioaccumulation and a potential threat to human health. Furthermore, inefficient fruit and vegetable cleaners used to address this problem often cause secondary pollution [3]. For example, as a long-acting pesticide, lead arsenate has been widely used worldwide since it was first applied in the US in 1965 [4]. Lead is a common heavy metal contaminant that has a particularly harmful effect on the human nervous system, reducing motor function and nerve conduction [5]. In addition, lead can cause anemia, damage to the digestive system, and cardiovascular diseases [6].

Ling et al. prepared nano-magnesium oxide (MgO) stabilized on N-doped biochar (MgO@N-biochar), which resulted in an increased number of surface functional groups of the adsorbent. The surface of this material possesses abundant amino, hydroxyl, and carboxyl groups. Using this material, a Pb adsorption of 893 mg/g can be achieved with a short equilibrium time of 10 min [7]. In addition, Wang et al. designed a chitosan sponge with high sulfur and nitrogen contents, which overcomes the acid intolerance of chitosan, provides a new chelating group while retaining the free amino groups, and yields a Pb adsorption capacity of 188.04 mg/g [8]. Furthermore, Luo et al. found that increasing the exposure of oxygen and chlorine sites on the surfaces of layered two-dimensional iron oxychloride (FeOCl) nanosheets can significantly increase the adsorption of Pb(II). Based on this, they peeled off large pieces of FeOCl to obtain ultra-thin FeOCl nanosheets (U-FeOCl) with a chlorine-rich and oxygen-rich surface, which exhibited a maximum adsorption capacity of 709 mg/g [9].

Owing to the growth of marine mussel aquaculture, the production of a large number of abandoned mussel shells has become an issue [10], and their excessive accumulation can lead to environmental pollution [11]. In the area of Zhoushan, which is the main producing area of mussels in the country, the annual output of mussels is ~80,000 tons and the quantity of discarded shells is ~64,000 tons. If a large number of discarded shells are not treated for a long time, it may cause a foul smell because the remaining shell meat rots or the microorganisms decompose salt into gas, such as H_2_S, NH_3_, and amine. These problems will have a negative impact on the quality of life of residents nearby and lead to environmental pollution [12]. To prevent environmental pollution and address the issue of resource wastage, the utilization of discarded mussel shells is particularly desirable. Indeed, the production and processing process of calcined mussel shells is rapid, convenient, simple, and easy to industrialize. The application of mussel shells in the adsorption of lead would, therefore, address the above environmental issues and also realize the reuse of mussel shell waste. In terms of their composition, the main component of mussel shells is calcium carbonate (CaCO_3_). They also contain a small amount of organic matter, such as protein and carbohydrates [13]. Mussel shells naturally have a porous surface structure, and shell powder is often used to treat sewage or to improve soil quality. In this context, Conde-Cid et al. studied the retention of sulfonamides and tetracyclines in the soil by mussel shells and found that the adsorption capacity was not significant [14,15]. However, Peña-Rodríguez et al. found that calcined mussel shells could remove 90% of mercury from water within 90 min [16]. In addition, Osorio-López et al. found that pyrite materials modified by mussel shells can significantly reduce the risk of arsenic (vanadium) pollution in the soil [17]. Furthermore, Garrido-Rodriguez et al. studied the adsorption and desorption ability of mussel shells toward cadmium, copper, nickel, and zinc in mining area soil using intermittent column experiments and found that mussel shells can significantly reduce the migration of heavy metals to the deeper regions while also reducing the risk of water pollution caused by heavy metal migration [17].

To date, little research has been conducted into Pb(II) adsorption by calcined mussel shells following simple calcination treatment. Thus, we herein report a novel usage of abandoned calcined mussel shells, wherein the waste mussel shells are crushed and calcined to yield a sorbent material, and the morphologies of the obtained particles are characterized. Using Pb(II) as a representative contaminant, the adsorption properties and the adsorption behavior of the shell powder are also evaluated. Overall, we aim to expand the fields of application of discarded mussel shell powder and promote resource utilization of discarded mussel shells.

## 2. Materials and Methods

### 2.1. Chemical Reagents

Pb(NO_3_)_2_ (AR) was purchased from Sigma-Aldrich (St. Louis, MO, USA), HNO_3_ (GR), NaOH (AR), methanol (AR), and HCl (AR) were purchased from Sinopharm Chemical Reagent Co., Ltd. (Shanghai, China). The discarded mussel shells were collected from Shengsi, Zhoushan Province, China.

### 2.2. Preparation of Calcined Shell Powder

The collected discarded mussel shells were cleaned and washed to remove the small amount of shell meat remaining in the shells. Subsequently, the shells were soaked in 0.5% dilute hydrochloric acid for 30 min to remove any impurities on the shell surfaces. After soaking, the shells were washed using purified water and dried. After cutting the shells roughly into small pieces, crushing using a multifunctional high-speed pulverizer (JP-300A, Yongkang Jiupin Industry and Trade Co., Ltd., Yongkang, China), they were sieved through 100-mesh [18] and calcined at 1000 °C in a muffle furnace over 3 h. The resulting shell powder was used for subsequent experiments [19].

### 2.3. Characterization of the Calcined Shell Powder

The morphology of the as-prepared calcined mussel powder was investigated by scanning electron microscopy (SEM; QUANTA FEG 400, FEI, Hillsboro, OR, USA). X-ray diffraction (XRD; D8 Advance, Bruker, Germany, voltage 40 kV, current 40 mA, step length 0.02°, test speed 0.1 s/step, λ(Cu) = 0.15418 nm) was used to analyze the crystal phases of the mussel shell powder before and after calcination, and differential scanning calorimetry (DSC; Q2000, American TA Company, New Castle, DE, USA, N_2_, 0–600 °C, 10 °C/min) was performed to examine the thermal behavior of the mussel shell powder. The porosities and specific surface areas of the mussel shells before and after calcination were measured using a Brunauer–Emmett–Teller (BET) analyzer (BK122T-B, JWGB, Beijing, China). The Zetasizer Nano instrument (Malvern Instruments, Malvern, UK) was used to measure the zeta potential of the calcined mussel shell powder at different pH. In addition, the shell powder samples before and after calcination were dried at 70 °C and characterized by a Fourier Transform Infrared (FTIR) spectrometer (Bruck Tensor II FTIR, Bruker, Germany) with a resolution of 4 cm^−1^ and a scanning range of 400–4000 cm^−1^, using the KBr particle method. X-ray photoelectron spectroscopy (XPS; Thermo Fisher Scientific, Waltham, MA, USA; ESCALAB 250Xi, Monochrome Al Kα, hv = 1486.6 eV, power 150 W, 500 μm beam spot) was used to investigate the surface elemental compositions of the calcined mussel powder before and after Pb adsorption [20].

### 2.4. Adsorption of Pb(II)

A 1 mg/mL lead standard solution was prepared using deionized water as the stock solution [21]. This stock solution was diluted to give different concentrations of aqueous Pb(II) solutions (i.e., 100, 80, 60, 40, and 20 mg/L) which were placed in 100 mL conical flasks. The desired quantity of calcined mussel shell powder was then added, and the adsorption test was performed at 150 rpm/min. We studied the effects of the amount of calcined mussel shell powder (10–50 mg), the pH (4–6), the temperature (293–308 K), and the equilibrium time on the adsorption capacity and Pb(II) removal rate during the entire adsorption process. After adsorption, the shell powder was removed by centrifugation, and the lead content was detected by flame atomic absorption spectrophotometry (AAS, AA-7000, Shimadzu Co., Ltd., Kyoto, Japan). The adsorption capacity and removal rate were calculated according to the following equations [22]:

(1) $$q_{e}=\left(C_{0}-C_{e}\right)\times \frac{V}{m},$$

(2) $$q_{t}=\left(C_{0}-C_{t}\right)\times \frac{V}{m},$$

where $q_{e}$ (mg/g) is the adsorption capacity of the calcined mussel shell powder toward lead at equilibrium, $q_{t}$ is the adsorption capacity at time *t* (mg/g), $C_{0}$ (mg/L) is the lead concentration in the solution prior to adsorption, $C_{e}$ (mg/L) is the lead concentration in the solution at equilibrium, and $C_{t}$ (mg/L) is the remaining lead concentration in the solution at time t; the volume of the lead solution matched by each adsorbent for each adsorption is recorded as *V* (L), and the adsorbent dose corresponding to each adsorption is recorded as *m* (g).

### 2.5. Desorption Studies

Following the adsorption of Pb(II) by the calcined mussel shell powder, separation of the powder was carried out by suction filtration. The separated adsorbent was then dried and mixed with EDTA solution (20 mL, 0.1 mol/L), shaken, and allowed to desorb for 8 h. After this time, the supernatant was removed, and the Pb(II) concentration was measured by AAS. The Pb(II) desorption experiment was then repeated using the same batch of calcined mussel shell powder for a total of 4 cycles, and the adsorption capacity of the pellets calculated after each desorption cycle.

### 2.6. Standard Deviation Calculations

The test data obtained were obtained by repeating in triplicate as a minimum. The standard deviation was calculated as outlined in Equation (3):

(3) $$S=\sqrt{\frac{\sum (Xi-\bar{X})}{n-1}},$$

## 3. Results and Discussion

### 3.1. Characterization

#### 3.1.1. FTIR Analysis

**Figure 1:**
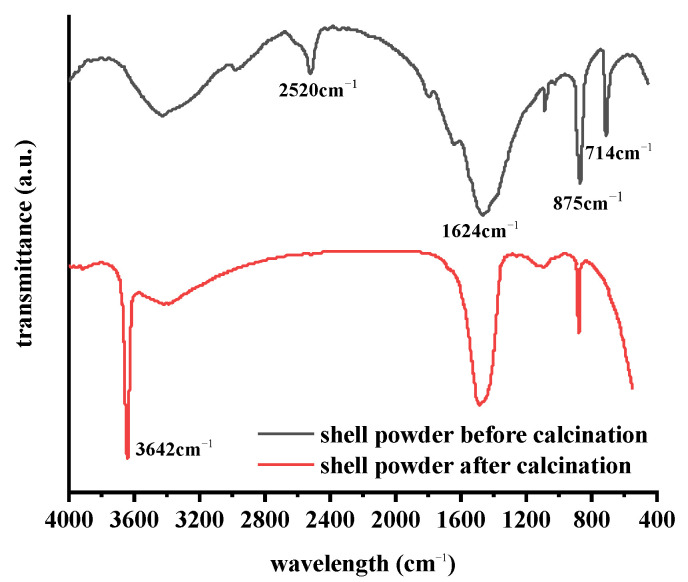
Fourier transform infrared (FTIR) spectra of the shell powder before and after calcination.

The shell powder was examined by FTIR before and after calcination, and the results are presented in Figure 1. As indicated, the sample before calcination showed clear absorption bands at 875 and 1426 cm^−1^, corresponding to the stretched and folded bands of the carbonate moiety [23]. In addition, 1426 cm^−1^ is the characteristic absorption peak of calcite, while those of aragonite can be observed at 875 and 714 cm^−1^, respectively [24]. These results indicate that prior to calcination, the main components of mussel shells were calcite and aragonite [25]. Other signals at 714 and 3600 cm^−1^ corresponded to the asymmetric bending vibration of carbonate rock and the tensile vibration peak of free OH^−^, respectively. It is evident from the spectrum that the absorption peak at 3642 cm^−1^ becomes stronger after calcination, and the sharp and narrow peak was considered to be the characteristic peak of CaO [26], indicating that the majority of calcium carbonate was decomposed after calcination. The band at 2520 cm^−1^ was considered to be the overtone of the carbonate band. In the calcined spectrum, the bands at 875 and 1426 cm^−1^ became weaker, and the band corresponding to the –OH part appeared near 3600 cm^−1^ [27], thereby indicating that the composition of the shell powder had changed. Therefore, the FTIR results showed that the mussel shell changed from calcium carbonate to calcium oxide after calcination. Moreover, the longer the calcination time, the more the amount of sample decomposed; the carbon dioxide produced by the decomposition of calcium carbonate may form a porous structure.

#### 3.1.2. DSC Analysis

**Figure 2:**
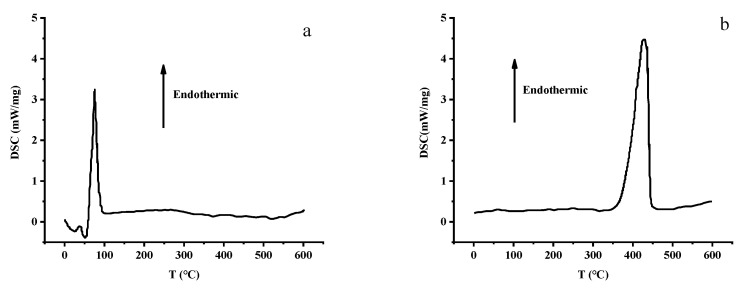
Differential scanning calorimetry (DSC) traces of the shell powder (**a**) before, and (**b**) after calcination.

DSC was used to study the thermal behavior of the shell powder. The results are shown in Figure 2.

Prior to calcination, a clear peak at 100 °C was observed in the DSC trace of the shell powder (Figure 2a), which originated from the vaporization of water adsorbed in the sample. In contrast, after calcination, a peak was observed at 400 °C (Figure 2b), which was attributed to a phase change at this temperature. Indeed, it is known that the phase transition of aragonite tends to take place between 250 and 400 °C. Since the organic matter and water molecules (crystal water) in the shell form hydrogen bonds with the oxygen atoms of carbonate ions in the plane, phase transitions may occur. When the shell powder is heated, the hydrogen bonds will be broken [28], steam will inevitably be generated, and the volume of small pores formed by the release of CO_2_ will increase. This produces a calcined mussel powder with a sufficiently porous structure for use as an adsorbent. These changes in the crystal structure upon calcination indicate the conversion of calcium carbonate to calcium oxide, as supported by the XRD results.

#### 3.1.3. XRD Analysis

**Figure 3:**
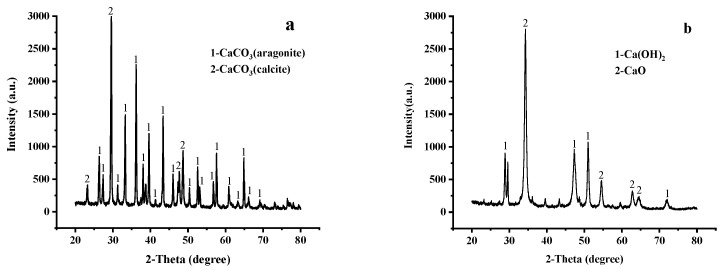
X-ray diffraction (XRD) patterns of the shell powder (**a**) before, and (**b**) after calcination.

The XRD patterns of the shell powders before and after calcination are shown in Figure 3.

As indicated in Figure 3, the composition of shell powder changed significantly during calcination. More specifically, as shown in Figure 3a, which is the XRD pattern of the shell powder before calcination, the main diffraction peak was observed at 2θ = 29°, and corresponds to calcite. Diffraction peaks of calcite and aragonite were also observed at 2θ = 25, 30, 34, and 54° [29]. As shown in Figure 3b, after calcination, the main diffraction peaks were observed at 2θ = 29, 35, 47, 51, 55, and 65°, which are the characteristic peaks of calcium oxide [30]. After calcination at a high temperature, almost all calcium carbonate was converted into calcium oxide. However, CaO can easily react with H_2_O in the air to form Ca(OH)_2_. Therefore, the XRD spectrum of shell powder after calcination also shows the characteristic peak of Ca(OH)_2_ (Figure 3b) [31]. FTIR can help prove the conversion of calcium carbonate to calcium oxide.

#### 3.1.4. SEM Analysis

**Figure 4:**
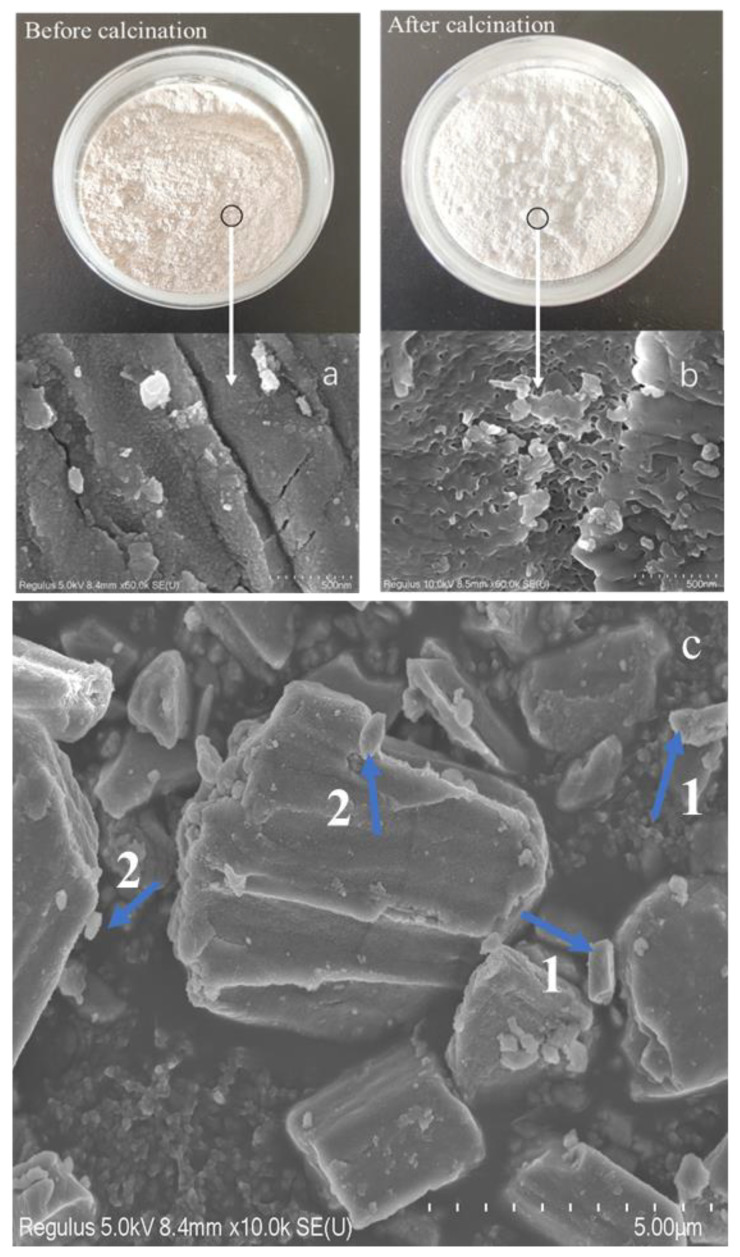
Scanning electron microscopy (SEM) images of the shell powder (**a**,**c**) before, and (**b**) after calcination.

The SEM images of the mussel shells before and after calcination are shown in Figure 4.

As shown in Figure 4a, before calcination, the shell powder particles exhibited a relatively dense structure with a relatively smooth surface. In Figure 4c, the rhombic calcite and lamellar aragonite structures are marked by arrows 1 and 2, respectively [32]. As can be seen from this figure, prior to calcination, the mussel shell powder was mainly composed of calcite and aragonite, while after calcination and activation (Figure 4b), the surface of the shell powder became rough, the structure became loose, and a large number of pores appeared. This structural change was the result of the decomposition of organic matter present in the shell powder and the volatilization of crystal water during high-temperature calcination, which ultimately results in the formation of a porous surface structure [33]. Such a porous structure can facilitate the loading of pollutants, such as heavy metal ions and pesticides, indicating that the calcined mussel powder may be a good adsorbent.

#### 3.1.5. XPS Analysis

**Figure 5:**
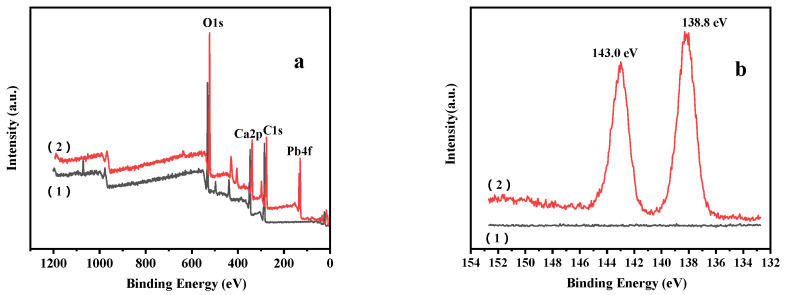
(**a**) X-ray photoelectron (XPS) wide scan, and (**b**) Pb 4f spectra of the shell powder (1) before, and (2) after the adsorption of Pb(II).

We subsequently used XPS analysis to elucidate the changes in the elements present on the surface of the calcined mussel powder before and after adsorption to confirm the successful Pb(II) adsorption. The results are shown in Figure 5.

Figure 5a,b shows the wide-scan and Pb 4f spectra of the sample before (1) and after (2) adsorption, respectively. After the adsorption process, the characteristic peak of lead was observed, indicating that the shell powder had indeed adsorbed Pb(II). In addition, a characteristic peak at 138.8 eV corresponding to the Pb 4f 7/2 orbital appeared in the spectrum, and a peak at 143.0 eV corresponding to Pb 4f 5/2 orbital [34] was also observed, further confirming that adsorption had taken place. The broad XPS spectrum obtained after adsorption shows that the peak intensity of the corresponding O element on the adsorbent decreased significantly. It was, therefore, considered that mainly lead oxide and a small amount of lead carbonate were present after adsorption.

#### 3.1.6. BET Analysis

**Figure 6:**
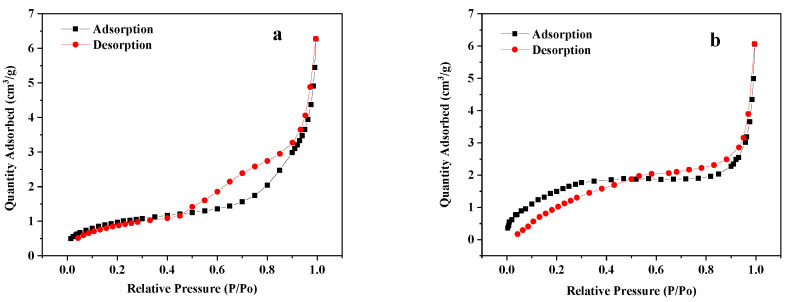
N2 adsorption and desorption analysis of the uncalcined mussel shells (**a**) and the calcined mussel shells (**b**).

Figure 6a,b displays the BET characterization results of mussel shells before and after calcination. The N_2_ adsorption-desorption isotherms of mussel shells before and after calcination belong to type III and IV pore models [35], respectively. It was evident that the mussel shell surface was multi-layered and almost non-porous before calcination, and the material after calcination had a mesoporous structure. The average pore diameter of the calcined mussel shells was 6.177 nm. The ionic radius of Pb^2+^ was 0.119 nm, which was smaller than the average pore diameter of the adsorbent. These results indicated that the calcined mussel shell had a higher specific surface area and porosity than the uncalcined sample and also had a higher adsorption efficiency.

### 3.2. Adsorption Testing

#### 3.2.1. pH

We added shell powder (0.03 g) to the Pb(II) solution (20 mL, 100 mg/L) at pH values of 4, 4.5, 5, 5.5, and 6 to investigate the effect of pH on the adsorption performance of the shell powder. The samples were agitated at 25 °C for a sufficient time to ensure that the adsorption equilibrium had been reached.

**Figure 7:**
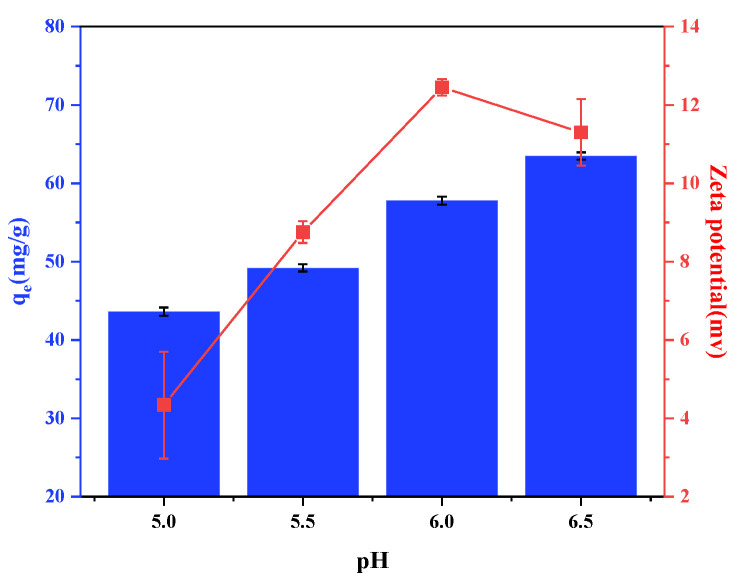
Effect of pH on the adsorption of Pb by the calcined mussel shell powder.

As shown in Figure 7, with the increase of pH, the adsorption capacity of the adsorbent increased from 43.62 mg/g to 63.49 mg/g. At low pH, there was less dissociation of Ca^2+^ in the solution, and Pb(II) exchanges with a small amount of Ca^2+^ in the solution; with the increase of pH, the degree of protonation decreased, and the dissociation of Ca^2+^ was more, Pb(II) replaced with more Ca^2+^, thereby increasing the adsorption capacity. In addition, the measurement results of Zeta potential on the surface of the material at different pH values showed that the surface of the material showed a positive potential under various pH conditions examined. It is believed that the surface of the material contained a large amount of Ca^2+^ dissociated from Ca(OH)_2_ and CaO. Therefore, when the pH was 6.0, the highest positive surface charge was recorded, leading to a greater degree of Ca^2+^ dissociation and stronger adsorption. When the pH value is further increased, Pb(OH)_2_ deposition will occur in the solution; therefore, pH 6.0 was considered to be the optimal pH for Pb adsorption after the calcination of mussel shell powder.

#### 3.2.2. Adsorbent Dosage

To examine the effect of the adsorbent dosage on the adsorption of Pb(II), we added 0.01, 0.02, 0.03, 0.04, and 0.05 g of shell powder to the 100 mg/L Pb(II) solution (20 mL), and the pH was adjusted to 6.0 using 0.01 M hydrochloric acid and 0.01 M sodium hydroxide. The resulting solution was shaken at 25 °C until equilibrium was reached.

**Figure 8:**
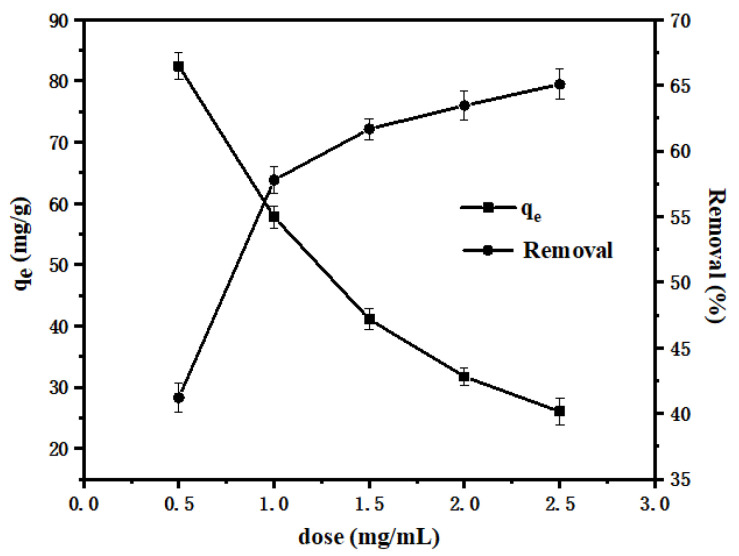
Effect of adsorbent dosage on the adsorption of Pb(II).

It can be seen from Figure 8 that as the amount of shell powder increased, the *q_e_* of the adsorbent gradually decreased from 82.42 to 26.03 mg/g, and the removal rate increased from 41.21% to 65.08%. This could be accounted for by considering the increased number of adsorption sites obtained by increasing the shell powder content. However, an excess of adsorbent was unnecessary because of the low concentration of Pb(II) in solution, which results in a low probability of all adsorption sites being occupied by Pb(II) [36]. Therefore, the adsorption capacity of the shell powder toward Pb(II) exhibited a downward trend upon increasing the adsorbent amount. Considering these results, we selected 1.0 mg/mL as the optimal adsorbent concentration for subsequent experiments.

#### 3.2.3. Comparison of the Adsorption Performance before and after Calcination

The calcined shell powder and the uncalcined shell powder were used to adsorb Pb(II) under the same adsorption conditions (20 mg shell powder, 100 mg/L Pb(II) initial concentration, 25 °C, and pH 6.0) to explore the effect of calcination on the adsorption of Pb(II). Prior to calcination, the adsorption capacity was 32.34 mg/g, while after calcination, this value increased to 57.79 mg/g, thereby indicating that calcination can effectively improve the adsorption performance of the shell powder toward Pb(II). This may be because of the decomposition of calcium carbonate into calcium oxide upon calcination, which allows carbon dioxide to be released, thereby creating a large number of small micropores. This was also consistent with the characterization results of the shell powder before and after calcination.

#### 3.2.4. Adsorbent Action Time and Kinetics

**Figure 9:**
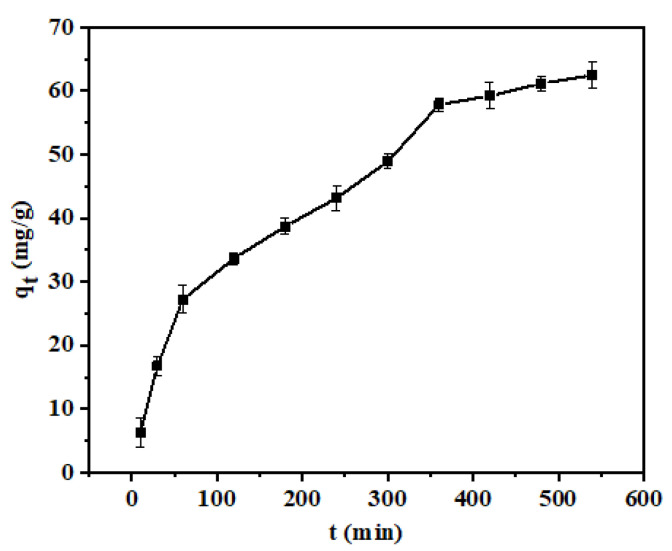
Effect of contact time on the adsorption of Pb(II).

To study the influence of the contact time on the adsorption of Pb(II), shell powder (0.02 g) was added to a solution of Pb(II) (100 mg/L), the pH adjusted to 6, and adsorption carried out at 25 °C for 10, 30, 60, 120, 180, 240, 300, 360, 420, 480, and 540 min (see Figure 9).

Figure 9 shows that within 360 min after the start of adsorption, the adsorption capacity increased rapidly for the calcined sample because of the presence of more abundant active sites. However, a further increase in the adsorption time only resulted in a slight increase in the adsorption capacity due to the gradual saturation of the calcined mussel shell powder [37]. The adsorption equilibrium was achieved at 540 min.

**Figure 10:**
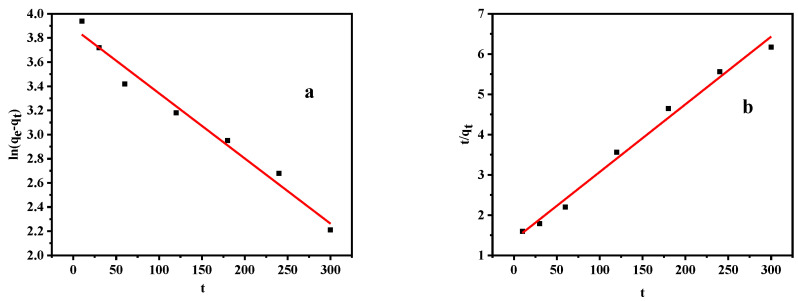
Fitting plots for the (**a**) pseudo first-order (PFO), and (**b**) pseudo second-order (PSO) models for the adsorption of Pb(II) on the calcined shell powder.

We then employed the pseudo first-order (PFO; Equation (3)) and pseudo second-order (PSO; Equation (4)) kinetic models to fit the adsorption data [38,39], as outlined in the following equations (see also Figure 10):

(4) $$q_{t}=q_{e}(1-e^{{-k}_{1}t}),$$

(5) $$q_{t}=\frac{q_{e}^{2}k_{2}t}{1+k_{2}q_{e}t},$$

where *k*_1_ (min^−1^) is the constant of the PFO model, and *k*_2_ (g/(mg·min)) is the constant of the PSO model.

Figure 10 shows the plots for the two kinetic models, while Table 1 outlines the kinetic parameters. A comparison of the R^2^ values indicates that the adsorption process was more in line with the PSO model. The calculated *q_e_* obtained by this model was 59.52 mg/g, which was similar to the experimental value (62.48 mg/g) [27]. Therefore, we believed that the adsorption of Pb(II) on the shell powder may be controlled by chemical adsorption [16]. As mentioned above, the calcined mussel shell powder contains large amounts of calcium oxide and calcium hydroxide. When placed in wastewater containing Pb, the Pb(II) ions can replace the Ca(II) ions present in calcium oxide and calcium hydroxide to complete the process of chemical adsorption.

#### 3.2.5. Temperature and Thermodynamics

**Figure 11:**
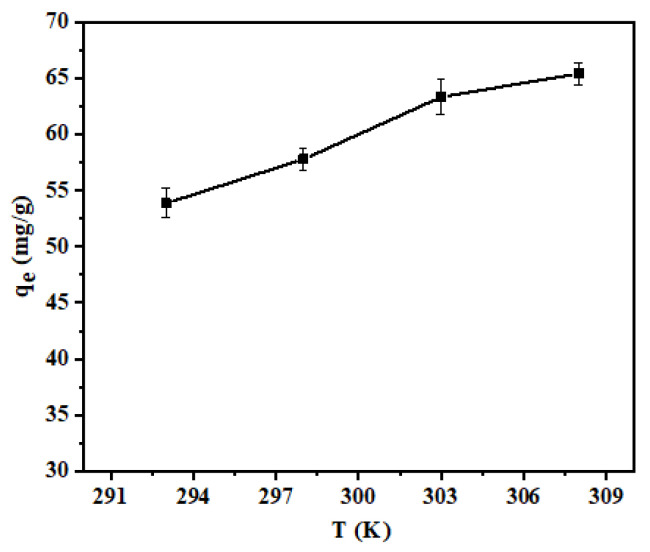
Effect of temperatures on the adsorption process.

To investigate the effect of temperature on the adsorption performance of the adsorbent, shell powder (0.02 g) was added to a Pb(II) solution (20 mL, 100 mg/L) at pH 6, and the sample was agitated until equilibrium was reached at 293, 298, 303, and 308 K (Figure 11).

It can be seen from Figure 11 that the adsorption capacity increased when the temperature was high; however, this increase was small. More specifically, the *q_e_* of the calcined shell powder for Pb(II) adsorption increased from 53.86 to 65.41 mg/g as the temperature was increased from 293 to 308 K. This may be because of an increase in molecular motion at higher temperatures, resulting in an increased probability of Pb(II) contacting the adsorption sites [40].

Using standard equations (Equations (6) and (7)), the thermodynamic correlation parameters of the adsorption process were calculated [41].

(6) $$\Delta G=-RT\ln\frac{q_{e}}{C_{e}}$$

(7) ΔG=ΔH−TΔS

where *R* (J/mol∙K) is the gas constant, and *T* (K) is the absolute temperature of the adsorption process.

**Figure 12:**
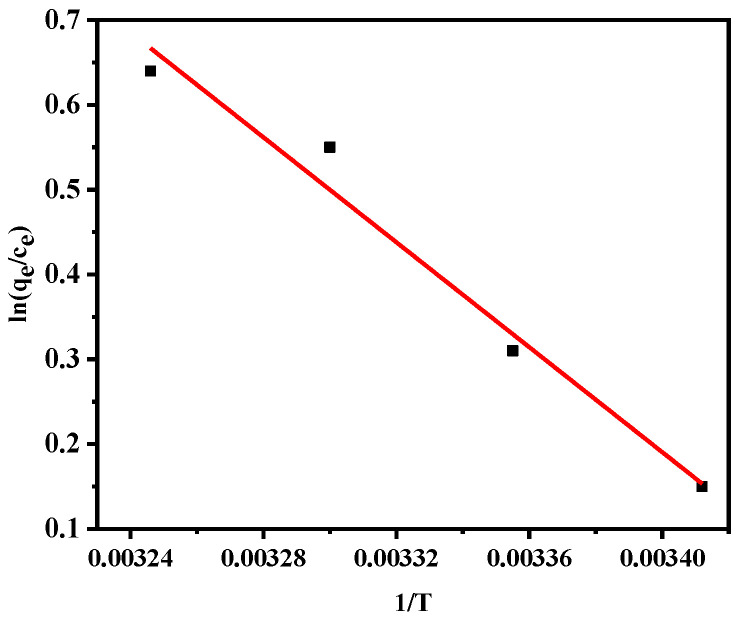
Plot of ln(*q*_e_/*C*_e_) versus 1/T for determination of the free energy change for the adsorption of Pb(II) by the calcined shell powder.

The Δ*G*, Δ*H*, and Δ*S* values are listed in Table 2, and a plot of the experimental data obtained using Equation (6) is shown in Figure 12. The negative Δ*G* value (Table 2) indicates that the adsorption process was endothermic and spontaneous [41]; therefore, increasing the temperature was beneficial for the adsorption process [42]. The positive Δ*H* and Δ*S* values also indicated that the process was endothermic [17,43].

#### 3.2.6. Initial Pb(II) Concentration and Isothermal Models

**Figure 13:**
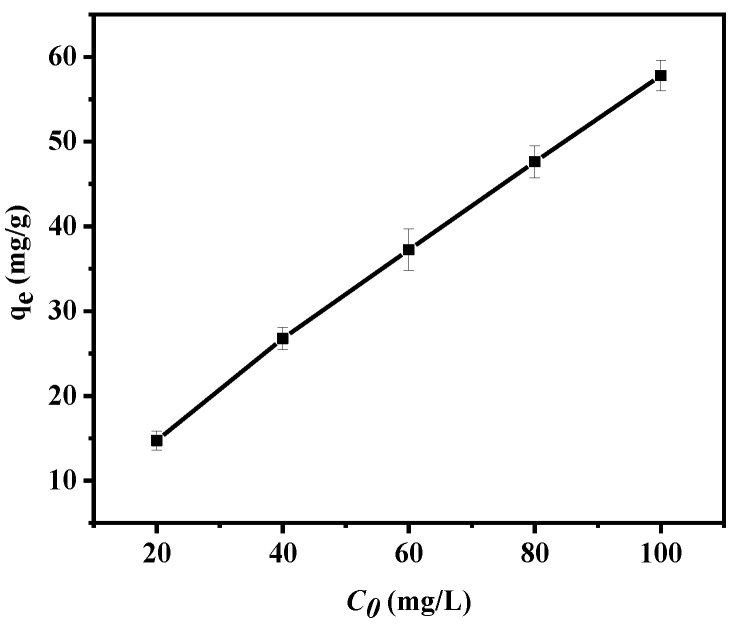
Plot of *q*_e_ versus *C*_0_ revealing the effect of the initial Pb(II) concentrations on the adsorption process.

Subsequently, the effect of the initial Pb(II) concentration on the adsorption performance was explored. Here, we used Pb(II) solutions (20 mL) at different initial concentrations (20–100 mg/L) to mix with the shell powder (0.02 g) in an Erlenmeyer flask and adjusted the pH to 6. The flask was agitated at 25 °C until the adsorption equilibrium was reached (see Figure 13).

As shown in Figure 13, upon increasing the initial lead concentration from 20 to 100 mg/L, the adsorption capacity of Pb(II) on the calcined shell powder gradually increased from 14.72 to 57.79 mg/g. At lower Pb(II) concentrations, the adsorbent did not reach saturation, while at higher concentrations, the available adsorption sites on the calcined shell powder surface gradually became occupied, and saturation was reached [17,44]. It should also be noted that increasing the Pb(II) concentration also provided a driving force for the resistance between the calcined shell powder and the heavy metal ions in the solution.

Overall, the calcined shell powder presented an excellent adsorption behavior toward Pb(II) ions. Thus, using the Langmuir and Freundlich isotherms (Equations (8) and (9), respectively), we analyzed the nature of the interaction between the shell powder and Pb(II) and obtained the maximum adsorption capacity of shell powder for Pb(II) [45,46]:

(8) $$q_{e}=\frac{q_{m}{bC}_{e}}{1+{bC}_{e}}$$

(9) $$q_{e}=K_{F}{C_{e}}^{n}$$

where *q_m_* (mg g^−1^) is the maximum adsorption capacity of the calcined shell powder, *n* is the heterogeneity factor, *b* is the constant of the Langmuir model, and *K_F_* is the constant of the Freundlich model.

The dimensionless constant of the Langmuir model (*R_L_*) was then calculated using Equation (10) to determine if the adsorption process is feasible [47].

(10) $$R_{L}=\frac{1}{1+bC_{0}}$$

**Figure 14:**
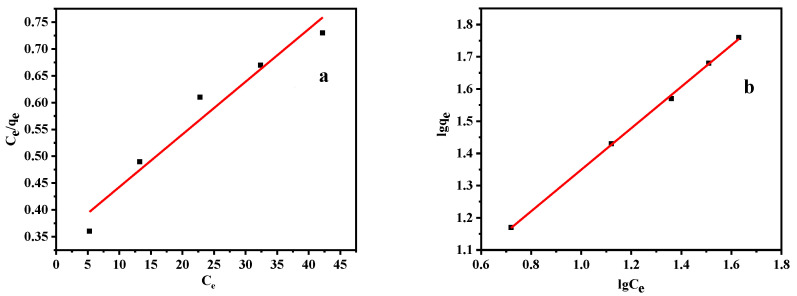
Fitting of the experimental adsorption data to the (**a**) Langmuir, and (**b**) Freundlich isotherms.

The calculated adsorption isotherm parameters are listed in Table 3, and the fitting curve of the experimental data is shown in Figure 14. By calculating the *R_L_* values for the Langmuir model, as shown in Table 4, *C*_0_ was observed to be within a range of 20–100 mg/L, 0 < *R_L_* < 1, meaning that the entire adsorption process can be studied. The R^2^ values of the Langmuir and Freundlich model fits were both determined to be >0.95, and the *q_max_* value obtained using the Langmuir isotherm was 102.04 mg/g, thereby further confirming that the calcined shellfish powder exhibited great potential for Pb adsorption. Following the fitting of the Freundlich isotherm model, an *n* value of 1.55 was determined (when the adsorption is slightly inhibited at a lower equilibrium concentration, 1/*n* < 1) [48], once again demonstrating that the calcined shell powder can adsorb Pb(II) ions. Moreover, based on the R2 values of the fitted models, the whole adsorption process was a better fit to the Freundlich isotherm, which may reflect the contribution of the porous structure of the calcined shellfish powder in the adsorption process. Both monolayer adsorption and physical adsorption were involved, thereby indicating that chemical adsorption and physical adsorption cooperate to achieve the adsorption of Pb(II) [49].

#### 3.2.7. Desorption Studies

**Figure 15:**
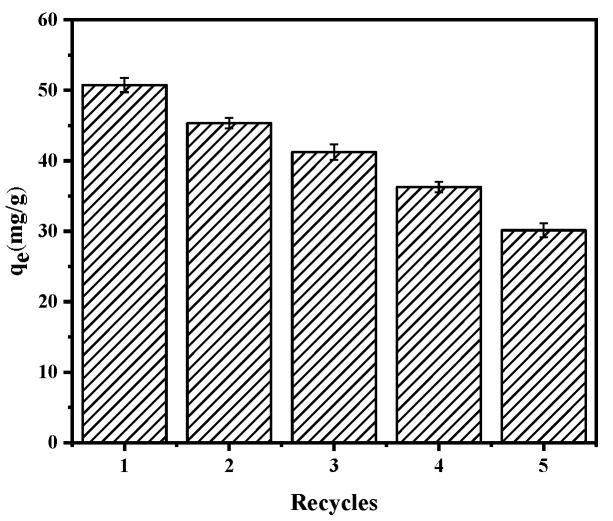
Recycling performance of the calcined mussel shells for Pb(II) adsorption.

Desorption studies were then carried out, as shown in Figure 15. Upon increasing the number of recycles to 5, the adsorption capacity of the shell powder toward Pb(II) was reduced, dropping from 50.76 to 30.14 mg/g; however, this was still considered a good adsorption performance.

#### 3.2.8. Comparison with Other Adsorbents

Finally, we compared the Pb(II) adsorption capacity of the calcined mussel shells to other reported adsorbents. As aforementioned, the maximum adsorption capacity obtained for the calcined mussel shells was 57.79 mg/g. Therefore, the results in Table 5 show that compared with some other adsorbents, the adsorption capacity of calcined mussel shells for Pb(II) ions was moderate. However, its raw material sources were sufficient, environmentally friendly, and economical, thus confirming its good application potential.

## 4. Conclusions

Based on X-ray diffraction, differential scanning calorimetry, and Fourier transform infrared spectroscopy analysis of the mussel shell powder before and after calcination, we determined that the crystal structure of the shell powder changed from the original calcite and aragonite phases to calcium oxide during the calcination process. In addition, scanning electron microscopy shows that the organic matter in the shell powder decomposes during the high-temperature calcination process to form a porous structure on the surface. In addition, it was inferred from the BET test results before and after calcination that the specific surface area of the calcined mussel shell powder increased significantly, and the surface pores were considerably larger than the Pb^2+^ diameter. Moreover, it was concluded that physical adsorption could be included in the adsorption process. X-ray photoelectron spectroscopy confirmed that the shell powder effectively adsorbed Pb(II) ions. Therefore, the adsorption performance of Pb(II) under different adsorption conditions was analyzed, and the optimal conditions were determined. The study of adsorption kinetics shows that the PSO kinetic model is the most suitable for the adsorption of Pb(II) on calcined mussel shell powder. At equilibrium, the Freundlich isotherm model is fitted to obtain a maximum adsorption capacity of 57.79 mg/g. In addition, using the Langmuir isotherm model, the adsorption capacity is calculated to be 102.04 mg/g. The R^2^ of the Freundlich and Langmuir isotherm models after fitting are both greater than 0.95, but the R^2^ value of the Freundlich model is larger. We believe that the entire adsorption process is more in line with the Freundlich isotherm model. In fact, the entire adsorption process involves both single-layer adsorption and multi-layer adsorption, and both isothermal models can describe the adsorption process well. Thermodynamic studies have shown that the interaction between the lead and the adsorbent involves a spontaneous endothermic reaction. Compared with other adsorbents, calcined mussel shell powder exhibits a better lead adsorption effect without secondary pollution. Overall, this study shows that calcined mussel shell powder can remove lead ions from aqueous solutions, and the reuse of mussel shells in such processes can greatly reduce the burden on the environment due to their continued shelving.

## Figures and Tables

**Table 1:** Kinetic parameters for the adsorption of Pb(II) on the calcined shell powder.

*q_e,exp_*	Pseudo First-Order (PFO)			Pseudo Second-Order (PSO)		
	*q_e,cal_*	*k*_1_	*R* ^2^	*q_e,cal_*	*k*_2_	*R* ^2^
62.48	48.51	0.0054	0.9780	59.52	0.0168	0.9895

*q_e,exp_*: Experimental *q_e_* (mg/g); *q_e,cal_*: Calculated *q_e_* (mg/g).

**Table 2:** Thermodynamic constants of the adsorption process for the calcined shell powder.

Concentration (mg/L)	Δ*G* (kJ/mol)				Δ*H* (kJ/mol)	Δ*S* (J/mol)
	*T* (K)					
	293.2	298.2	303.2	308.2		
100	−0.36	−0.81	−1.26	−1.70	25.73	89.06

**Table 3:** Parameters obtained from the Langmuir and Freundlich models for the adsorption of Pb(II) on the calcined shell powder.

*T* (K)	Langmuir			Freundlich		
	*q_max_^a^*	*B*	*R* ^2^	*K_F_*	*N*	*R* ^2^
298.2	102.04	0.028	0.9519	5.07	1.55	0.9992

*q_max_^a^* is the maximum adsorption capacity.

**Table 4:** *R_L_* values determined from the Langmuir model.

*C*_0_	*R_L_*
20	0.64
40	0.47
60	0.37
80	0.31
100	0.26

**Table 5:** Comparison of the maximum Pb(II) adsorption capacity of calcined mussel shells with previously reported adsorbents.

Adsorbent	*q_m_* (mg g^−1^)	Reference
Sulfonated lignin	123	[50]
Hydroxyapatite nanopowders	99.30	[51]
Chitin/lignin hybrid material	91.74	[52]
Calcined mussel shells	57.79	This work
Modified beer lees	29.6	[53]
Biochar	22.3	[54]
Spent coffee grounds	13.6	
Sludge-filter sand	20.41	[55]
Phragmites biomass	5.46	[56]
